# Seromolecular study on the prevalence and risk factors of *Toxoplasma gondii* infection in pregnant women referred to a gynecology hospital in Urmia, northwest part of Iran in 2022

**DOI:** 10.1186/s12879-024-09265-5

**Published:** 2024-04-17

**Authors:** Nasim Bashour, Arash Aminpour, Shabnam Vazifehkhah, Rasool Jafari

**Affiliations:** 1 Department of Parasitology and Mycology, School of Medicine, Urmia University of Medical Sciences, Urmia, Iran; 2 Department of Obstetrics and Gynecology, School of Medicine, Urmia University of Medical Sciences, Urmia, Iran; 3 Cellular and Molecular Research Center, Cellular and Molecular Medicine Research Institute, Urmia University of Medical Sciences, Urmia, Iran

**Keywords:** Toxoplasma gondii, Acute infection, Genotype, Pregnant women, Iran

## Abstract

Toxoplasmosis is a frequent infection among the human population. The infection can cause devastating complications for the fetus during pregnancy. The present study aimed to determine the serological and molecular prevalence of the infection and molecular characterization of *Toxoplasma gondii* isolates among pregnant women referred to Kowsar Hospital, Urmia, Iran. In a cross-sectional study, 340 blood samples were collected from pregnant women referred to Kowsar Hospital, Urmia, Iran from May to July 2022. Anti-*T. gondii* IgG and IgM seropositivity were determined by enzyme-linked immunosorbent assay. PCR was carried out by targeting the GRA6 gene of the parasite on all patients’ buffy coats. Anti-*T. gondii* IgG and IgM antibodies were positive in two (0.6%) women, and 101 (29.7%) women had anti-*T. gondii* IgG and 70.3% were seronegative. PCR was positive in two IgM-positive women, and both isolates belonged to *T. gondii* carrying the GRA6 allele of lineage I. The risk of infection was significantly higher in women who had constant contact with cats and soil, and who were residents of rural areas. The two IgM-positive women were asymptomatic regarding acute toxoplasmosis. According to the results of the present study, the prevalence of toxoplasmosis in pregnant women in Urmia is similar to its prevalence in other areas in northwestern Iran, and despite the low prevalence of acute infection, it should not be ignored.

## Introduction

*Toxoplasma gondii* is an intracellular protozoan parasite with a variety of intermediate hosts that infects mammals and birds [1]. There are two stages in the life cycle of this parasite, the intestinal stage in the final host and the extraintestinal stage in intermediate hosts such as humans and other warm-blooded animals [2]. Transmission of *T. gondii* occurs mainly by ingestion of the oocysts excreted by cats or by consuming raw or undercooked meat of infected animals containing tissue cysts. Tachyzoites of the parasite may pass through the placenta of the infected pregnant women and infect the fetus causing congenital toxoplasmosis [3].

Acute infections with *T. gondii* that occur during pregnancy may sometimes cause congenital infection which may have severe complications such as fetal death, miscarriage, and ocular, neurological, and other organ damage in the fetus. If the infection occurs in the early stage of pregnancy, the transmission rate is low, yet if the fetus is infected in this period, the chance of severe infection is high. *T. gondii* infections that occur after birth are usually asymptomatic [4]. It is estimated that *T. gondii* infects approximately two billion humans worldwide, while only a very small percentage of infected individuals develop serious disease [5]. In immunocompromised patients, acute infection or activation of chronic infection may cause serious complications such as encephalitis [6].

*T. gondii* is divided into three main genetic lineages, types I, II, and III, which are different in terms of pathogenicity and epidemiological status. In South America, some lineages are distinct and non-type I, II, or III, which were named ‘atypical ’ or ‘exotic’ types. Type I is lethal to mice, yet types II and III are considerably less virulent. In humans, type II is the principal lineage causing toxoplasmosis. Nevertheless, type I or type I-like atypical isolates are more likely to severely cause retinochoroiditis in human patients. The atypical isolates are often reported to cause severe disseminated acute toxoplasmosis in patients with healthy immune systems [7]. Most of the strains isolated from patients with AIDS belong to type II. Types I and II strains have been recorded in patients with congenital infection [8]. In Iran, based on the reports from human and non-human samples, Type III had the highest frequency followed by Type I and then Type II. Mix and atypical types had the lowest frequency [9].

The diagnosis of *T. gondii* infection in humans is generally achieved by serological methods [10]. These methods are commonly intended to detect anti-*Toxoplasma* IgG and IgM. Although a positive IgM *T. gondii* antibody result indicates an acute infection, IgM can remain for several months. A positive *T. gondii* IgM and IgG result could not interpreted as an infection that recently occurred [11]. Molecular diagnosis, however, has significant advantages in the detection of toxoplasmosis [12].

Considering that a comprehensive study using serology and PCR together with an emphasis on diagnosing the acute form of the infection has not been conducted in West Azerbaijan Province, the present study aims to determine the prevalence of acute and chronic toxoplasmosis using serological and PCR methods and determine the genotype of the parasite in pregnant women referred to Kowsar Hospital in Urmia.

## Materials and methods

### Study area and sample collection

This cross-sectional survey was carried out in Urmia, the capital of West Azerbaijan Province, North West Iran. The study was approved by the Ethics Committee of Urmia University of Medical Sciences under the Ethical Code of IR.UMSU.REC.1401.031. Participants were informed about the study and a signed informed consent was obtained from them.

Blood of 340 pregnant women of all gestational ages referred to Kowsar Hospital was collected during two months from May to July 2022. Blood samples were taken from pregnant women and divided into two parts; one for serum separation and the other for buffy coat isolation. The sera were separated from whole blood, and buffy coats were isolated by centrifugation of blood containing anti-coagulant and kept frozen at -70 °C until examination.

### Serological assays

ELISA was carried out on the collected sera for anti-*T. gondii* IgG and IgM antibodies, using commercial kits (Pishtazteb, Iran) following the manufacturer’s instructions. The anti-*Toxoplasma* IgG indirect ELISA kit was used with a sensitivity of 100% and specificity of 99% as claimed by the company. The kits contained standards (10, 50, 100, and 200 IU/mL) positive and negative controls. IgG antibody concentrations ​​higher than 11 were considered positive, ​​those between 9 and 11 were considered borderline, and those less than 9 were considered negative. The qualitative antibody capture anti-*Toxoplasma* IgM ELISA kit was 100% sensitive and 99% specific as claimed by the company. The kit contained three controls (negative, positive, and cut-off controls). Seropositivity was calculated based on the cutoff controls as follows. Cutoff index = sample OD/mean OD of cutoffs. An index higher than 1.1 was considered positive, less than 0.9 was considered negative and between 0.9 and 1.1 was considered borderline.

### DNA extraction and PCR

The genomic DNA from all 340 buffy coat samples of the pregnant woman was extracted using a commercial DNA extraction kit (NodexPlus, NAT Biotech, Iran) according to the instructions of the kit. The PCR test was performed using the GRA6 gene of *T. gondii* that was previously described as a nested PCR; however, in the present study, the inner primers targeting 344 bp sequence with annealing at 55 °C were used (F: TTTCCGAGCAGGTGACCT and R: TCGCCGAAGAGTTGACATAG) [13]. PCR was performed in a final volume of 20 µL, including 10 µL of premix (Ampliqon, Denmark), 1 µL of forward primer and 1 µL of reverse primer, 2 µL of template DNA, 4 µL of Q-solution and 2 µL of distilled water. The *T. gondii* RH strain and nuclease-free water were used as the positive and negative controls, respectively.

The thermal cycles were as follows. An initial denaturation at 95 °C for 5 min, 35 cycles of denaturation at 95 °C for 30 s, annealing at 55 °C for 30 s, extension at 72 °C for 30 s, and a final extension at 72 °C for 5 min. Then gel electrophoresis was carried out on the PCR products in a 1.5% agarose gel in TBE buffer.

### Sequencing

PCR-positive samples were reamplified in a volume of 50 µl and sequenced by the Sanger sequencing method with the forward primer. The obtained sequences were initially visually checked for any noise and low-quality peaks using SnapGene software v. 5.3.1 (available at www.snapgene.com), imported into MEGA11 (Tamura, Stecher, and Kumar 2021) [14], edited on both sides and aligned using ClustalW. The sequences were then compared with sequences from GenBank for closest genetic relatives and then deposited to the GenBank. A phylogenetic tree was built by the maximum likelihood method using the Tamura–Nei model (MEGA11) [15].

#### In silico restriction fragment length polymorphism (RFLP)

In silico RFLP simulation was performed in SnapGene software using *Tru1I* (*MseI*) restriction endonuclease. The 2.5% agarose gel electrophoresis was created and exported as an image file.

#### Data analysis

Data were analyzed by SPSS version 23 (IBM Corp. Released 2020. IBM SPSS Statistics for Windows, Version 27.0. Armonk, NY: IBM Corp) using chi-square, *t*- and logistic regression tests. *P* value < 0.05 was considered significant.

## Results

### Regional distribution

The studied population was referred from different regions of West Azerbaijan Province, which are listed in Table 1.

### Serology

Sera were collected from 340 pregnant women with an average age of 29.13 ± 6.65 years (16–46 years old) who were in different months of gestation. Out of 340 tested samples, both anti-*T. gondii* IgG and IgM antibodies were positive in two (0.6%) women and 101 (29.7%) women had anti-*T. gondii* IgG. *T. gondii* IgG seropositivity in different districts is available in Table 1.

*T. gondii* seropositivity was significantly higher in women who resided in rural regions (OR = 1.538; *P* = 0.049) and had contact with soil (OR = 2.043; *P* = 0.003 ) and cats (OR = 1.757; *P* = 0.014). The prevalence was lower in women who washed raw consumed vegetables with disinfectants, yet it was not statistically significant (OR = 0.417; *P* = 0.162). Additionally, no significant difference was found between *T. gondii* seropositivity and the consumption of undercooked meat (Table 1).

**Table 1:** Anti-*T. gondii* seropositivity among pregnant women considering different studied demographics and risk factors, in West Azerbaijan province, Northwest Iran. The odds ratio can be inversed by 1 ÷ OR

Variable			IgG		Total	OR	95% CI (OR)	*P*
			Positive	Negative				
**Residential**								
		Urban	56 (26.3%)	157 (73.7%)	213	0.650	0.404–1.045	0.049
		Rural	45 (35.4%)	82 (64.6%)	127	1		
**Cat contact**								
		Yes	45 (37.5%)	75 (62.5%)	120	1.757	1.089–2.834	0.014
		No	56 (25.5%)	164 (74.5%)	220	1		
**Soil contact**								
		Yes	68 (36.2%)	120 (63.8%)	188	2.043	1.256–3.326	0.003
		No	33 (21.7%)	119 (78.3%)	152	1		
**Meat consumption**								
		Undercooked	54 (32.1%)	114 (67.9%)	168	1.260	0.790–2.008	0.197
		Cooked	47 (27.3%)	125 (72.7%)	172	1		
**Vegetable wash**								
		Detergent	14(28.6%)	35 (71.4%)	49	-	-	0.332
		Salt and water	35(31.8%)	75 (68.2%)	110	1.167	0.558–2.441	0.682
		Water	48(31.4%)	105 (68.6%)	153	1.143	0.563–2.319	0.712
		Disinfectants	4(14.3%)	24 (85.7%)	28	0.417	0.122–1.421	0.162
District Name*								
		Urmia	83 (28%)	213 (72%)	296	-		0.303
		Showt	1	1	2			
		Salmas	4	7	11			
		Mahabad	2	4	6			
		Sardasht	2	2	4			
		Piranshahr	3	5	8			
		Poldasht	1	1	2			
		Khoy	4	1	5			
		Naghadeh	0	4	4			
		Bookan	1	1	2			
**Total**			101 (29.7%)	239 (70.3%)	340			

* **In the regional distribution, because of a low number of participants in some districts, the percentage would be invalid, thus it is not provided.**

Using the Kolmogorov‒Smirnov test, the normality of the data related to the age of the patients was checked, and the results showed that the data related to the age of the patients did not have a normal distribution (*P* < 0.001). Therefore, the nonparametric Mann‒Whitney U test was used to compare the average age among toxoplasmosis-positive and toxoplasmosis-negative people. The average age of the positive and negative studied women did not show any significant difference (Table 2).

**Table 2:** Comparison of mean age of toxoplasmosis-positive and toxoplasmosis-negative pregnant women in Urmia

IgG	N	Mean age	St. deviation	Mean rank	*P*
Positive	101	28.8	6.45	165.33	0.528
Negative	239	29.27	6.73	172.69	
Total	340				

The mean concentration of IgG in *T. gondii* IgG-positive people was 54.22 ± 2 IU/mL. The normality of these data was evaluated by the Kolmogorov‒Smirnov test and the results showed that the distribution of IgG concentration data in positive pregnant women was normal (*P* = 0.2). Therefore, the *t*-test and analysis of variance (ANOVA) were used to analyze the relevant data. Using the *t*-test, the average IgG concentration was not significantly different regarding different risk factors such as having contact with cats and soil, living in rural areas, and consumption of undercooked meat (Table 3).

**Table 3:** Comparison of the mean concentration of anti-*T. gondii* IgG of positive women with the studied risk factors

Variable		N	Mean IgG IU/mL	Std. Deviation	t	*P*
Contact with cat	Yes	45	50.1102	22.19971	1.690	0.588
	No	56	57.4880	21.48336		
Residential	City	56	55.4950	22.91698	0.657	0.578
	Rural	45	52.5904	20.96052		
Soil contact	Yes	68	54.7684	21.68117	0.370	0.331
	No	33	53.0315	22.95717		
Meat consumption	Undercooked	54	53.6057	21.13195	0.290	0.604
	Cooked	47	54.8847	23.18085		

ANOVA was used to compare the mean IgG concentration in variables with more than two variants. The results showed a lower concentration of anti-*T. gondii* IgG among patients who used disinfectants for washing vegetables compared to those who used water (*P* = 0.008) and salt (*P* = 0.016) (Table 4).

**Table 4:** Comparison of the mean concentration of anti-*T. gondii* IgG of positive women with different methods of washing vegetables

(I) Washing vegetable	(J) Washing vegetable	Mean Difference (I-J)	*P*	95% CI	
				Lower Bound	Upper Bound
Detergent	Salt	-4.34414	0.916	-21.8399	13.1516
	Water	-6.30211	0.761	-23.1073	10.5031
	Disinfectants	29.69393	0.070	-1.6732	61.0611
Salt	Detergent	4.34414	0.916	-13.1516	21.8399
	Water	-1.95797	0.976	-14.2555	10.3396
	Disinfectants	34.03807^*^	0.016	4.8368	63.2393
Water	Detergent	6.30211	0.761	-10.5031	23.1073
	Salt	1.95797	0.976	-10.3396	14.2555
	Disinfectants	35.99604^*^	0.008	7.2032	64.7889
Disinfectants	Detergent	-29.69393	0.070	-61.0611	1.6732
	Salt	-34.03807^*^	0.016	-63.2393	-4.8368
	Water	-35.99604^*^	0.008	-64.7889	-7.2032

* Significant

#### PCR

**Fig. 1:**
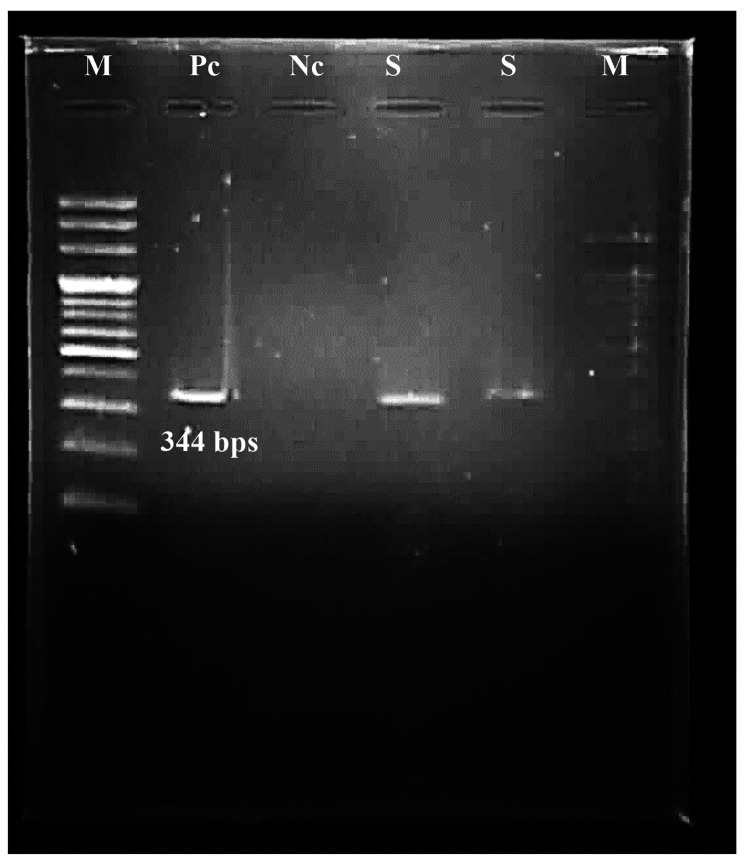
Gel electrophoresis of positive samples (S) along with positive control (Pc), negative control (Nc), and bp100 marker (M)

PCR was performed on all 340 blood samples of the studied pregnant women on the GRA6 gene (344 bp fragment). None of those women that were solely positive for anti-*T. gondii* IgG was positive in the PCR and only the two IgM-positive samples (0.6%) were found to be PCR positive (Fig. 1).

#### Sequencing

**Fig. 2:**
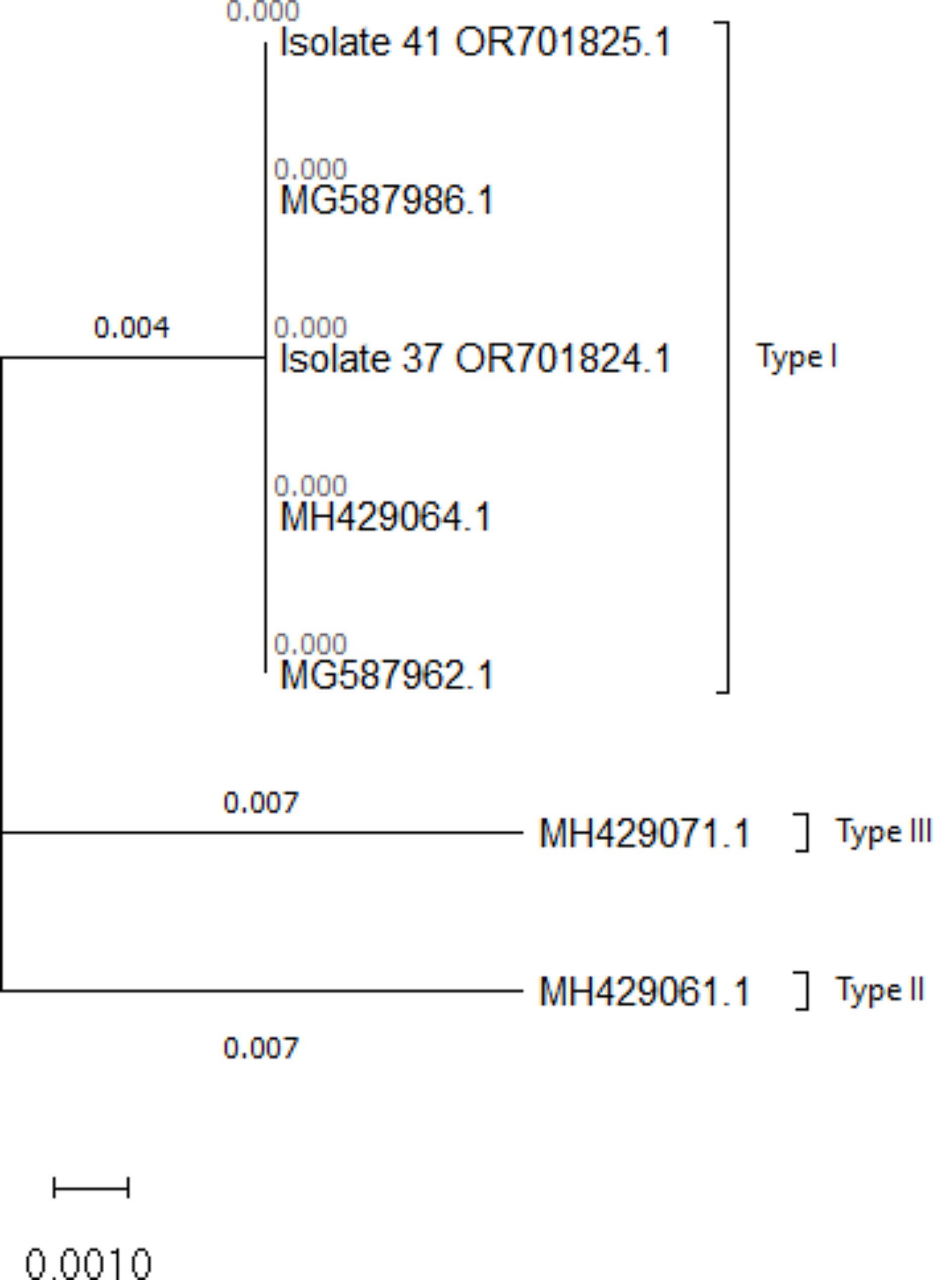
Phylogenetic analysis of two human isolates (pregnant women) of *T. gondii* using the 288 bp fragment of GRA6 gene sequence. The maximum likelihood method and Tamura-Nei model were used to infer the evolutionary history [15]. The phylogenetic tree with the highest log likelihood (-429.09) is illustrated in the figure. This analysis was carried out on the five nucleotide sequences. There were 288 positions in the sequences. Evolutionary analyses were conducted in MEGA11 [14]. The isolates (37 and 41) under the accession numbers of OR701824 and OR701825 are the isolates of the present study

After editing both sides of the two sequences, 288 bp remained. The sequences were then compared to the reference sequences of the GenBank and both were found to be 100% homologous with the type I of *T. gondii* (Fig. 2), however, because the sequence is short and there may be common sequences among different types, it could be a GRA6 allele of type I.

The sequences were deposited to the GenBank under the accession numbers of OR701824 and OR701825.

#### In silico RFLP

**Fig. 3:**
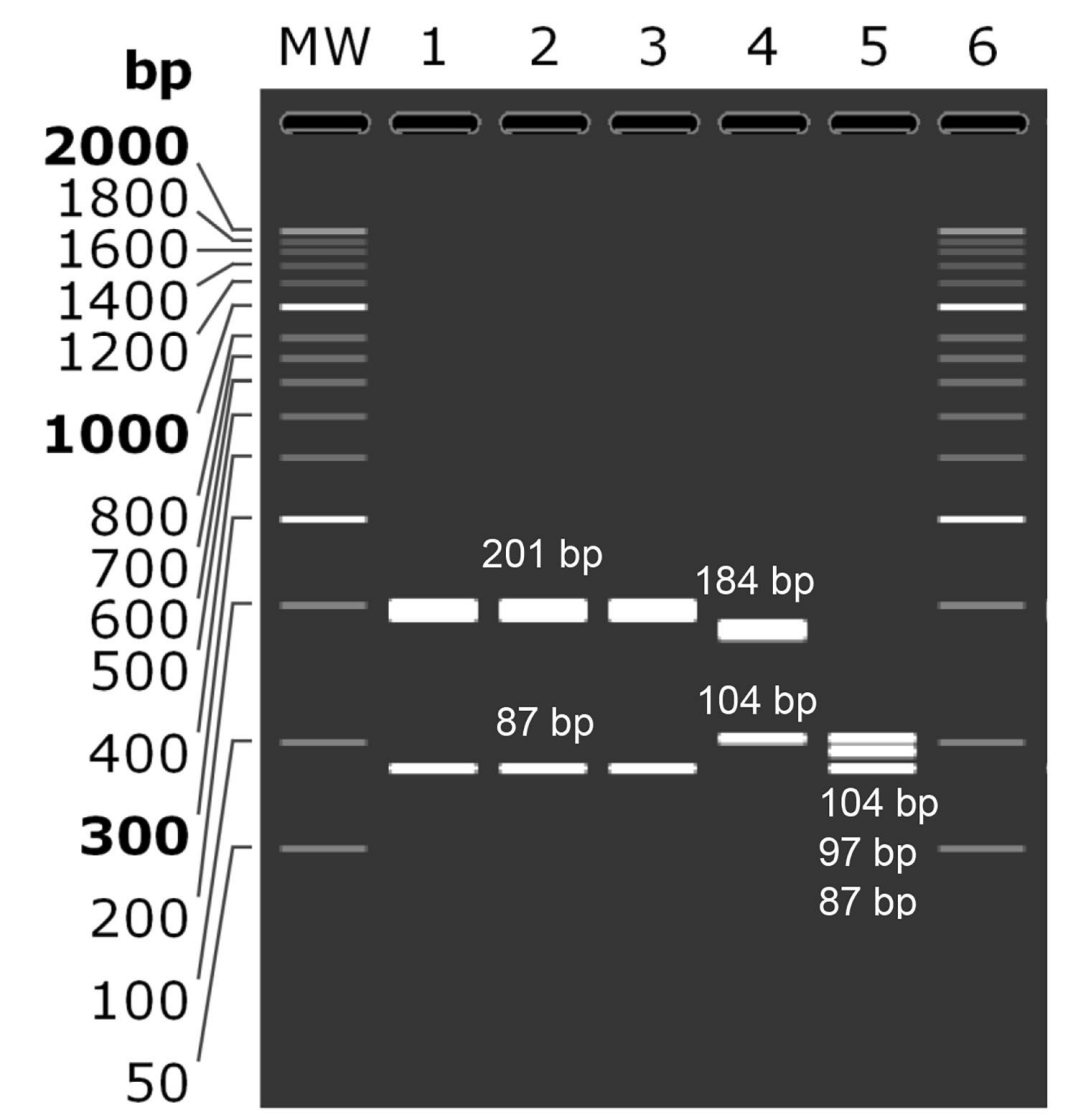
Software simulation of gel electrophoresis of the enzymatic digestion by endonuclease *Tru1I* (*MseI*) in the in silico RFLP on the 288 bp fragment of GRA6 gene of *T. gondii*. Lane MW and 6: 50 bp DNA marker; lane 1 (type I: MH429064.1), 4 (type II: MH429061.1), and 5 (Type III: MH429071.1): controls; lane 2 and 3: isolates of the present study

The sequences of two isolates each 288 bp and controls from the gene bank for each type I, II, and III of *T. gondii* were simulated in 2.5% agarose gel after being digested with *Tru1I* (*MseI*) endonuclease using SnapGene software. The endonuclease cleaved and produced two 201 and 87 bp fragments for type I, two 184 and 104 bp fragments for type II, and three 104, 97, and 87 bp fragments for type III (Fig. 3).

## Discussion

Toxoplasmosis is a zoonotic infection with a wide geographic distribution. Humans acquire the infection in different ways such as eating raw or undercooked meat containing tissue cysts (bradyzoites) or ingesting oocysts in contaminated soil, water, or food [16]. Infection in pregnant women can cause serious complications in the fetus such as miscarriage, mental retardation, and eye and nervous system damage [17].

In the present study, out of 340 blood samples of pregnant women that were tested, 29.7% had a positive level of IgG and 0.6% had a positive level of IgM against *T. gondii*. The highest infection rate was related to women who lived in rural areas. In a study conducted on pregnant women referred to Al-Zahra Medical Center in Tabriz in 2011, 26.3% of the studied pregnant women had a positive IgG titer and 0.33% had a positive IgM titer. *T. gondii* infection seropositivity was higher in pregnant women in contact with cats [18]. In another similar study conducted by Hazrati Teppeh et al. 2014 on pregnant women in Urmia, 28.32% of the studied population was anti-*T. gondii* IgG positive and 1.44% was IgM positive [19]. In our study, the IgG and IgM titers were similar to those in the two studies mentioned in Tabriz and Urmia.

Considering studies from different regions of Iran, the prevalence of toxoplasmosis in pregnant women was reported to be 31.1% in Tehran in 2010–2013 [20], 41.8% in Gorgan in 2012 [21], 32% in Yazd in 2012 [22], and 37.2% in Zanjan in 2012 [23]. The reported prevalence of toxoplasmosis in pregnant women is different among countries. For example, the prevalence of toxoplasmosis in pregnant women was reported to be 70.8% in northwest Ethiopia in 2019 [24], 70.8% in Brazil from October 2004 to April 2005 [25], and 55% in India from January 2005 to 2006 [26].

The most important factors involved in the spread of toxoplasmosis are geographical conditions, humidity level, heat, food habits, and hygiene practices [27]. The results of our study showed that there is no significant relationship between the consumption of undercooked meat and the method of washing vegetables with toxoplasmosis, which is different from the results of a report from Fallah et al. in 2004 in Hamedan, West of Iran [28]. In the present study, there was a significant relationship between contact with a cat and acquiring *T. gondii* infection, which is in contrast with the report of Cheraghipour et al. 2010 in Khorramabad, western Iran [29]. The results of our study showed that there is a significant relationship between being commonly in contact with soil and toxoplasmosis, which was consistent with the results of the study by Sharbat Khouri et al. in 2012 in Gorgan City, Golestan Province [21] and also in 2012 in Tabriz, a neighboring city [30].

In this research, molecular diagnosis and identification of *T. gondii* were performed by targeting the GRA6 gene in all 340 samples. In two cases that were IgM positive, the infection was also positive with PCR. In a study conducted by Turcekova and colleagues in 2012 in Kosice, 15 amniotic fluids and 1 blood sample from pregnant women suspected of toxoplasmosis were analyzed. The presence of *T. gondii* in the blood of a pregnant woman was confirmed and identified as genotype I, which is the same genotype identified in the present study in two pregnant women [31].

Jawahir Alghamdi et al. in Saudi Arabia in 2011, reported the seroprevalence of *T. gondii* IgG and IgM antibodies in 32.5% and 6.4% of pregnant women, respectively [32]. Contrary to the finding of the present study, in their report, 29 samples (80.6%) out of 203 pregnant women were genotype II, and 7 samples (19.4%) were genotype III [32]. Furthermore, *T. gondii* DNA was reported in 3.8% (8/210) of paraffin-embedded fetoplacental tissues of women with recurrent spontaneous abortion, which all positive samples belonged to type III [33], which is different from the genotype found in the present study (type I). Based on the review by Sadeghi et al. 2022, among the genotypes reported from Iran on human and non-human samples, type III had the highest frequency followed by Type I and then Type II [9].

In 2004 Asmar et al. performed a serological examination of sera from 200 pregnant women referred to the Department of Parasitology, Pasteur Institute of Iran, using IFA and reported positive serum antibody titers against *T. gondii* in 49 (24.5%) of their studied samples. Subsequently, nested PCR targeting the B1 gene was performed on 11 samples of amniotic fluid from women with anti-*T. gondii* IgM, of which four samples were found to be positive [34]. In the present study, amniotic fluid was not used, however, both IgM-positive women were also positive by PCR, and all of the solely IgG-positive ones were negative in the PCR.

In the study of Aref Khah et al., 100 tissue samples of spontaneously aborted fetuses and their mother’s blood were analyzed in Kohgiluyeh and Boyer Ahmed Province from 2015 to 2016. Mothers’ sera were examined for anti-*T. gondii* antibodies, while fetal tissues were examined for the presence of *T. gondii* DNA. They performed PCR targeting the GRA6 gene and then sequenced them. Seven percent of their studied women were IgG and 3% were IgM positive. Using real-time PCR on the buffy coat, one seronegative case and two IgM-positive cases (out of three cases) were also positive for *T. gondii*, which were related to genotype I, similar to the findings of the present study. In Arif Khah et al. study, as in our study, DNA was extracted from all samples and PCR was performed on all studied populations. In their study, a seronegative individual was positive by PCR [35]; however, in the present study, only IgM-positive individuals were also positive by PCR.

## Limitations of the study

We had two main limitations; first, we could not follow up with the two pregnant IgM-positive women to see whether after delivery their infant was healthy or with congenital toxoplasmosis. The second limitation was not using other genetic markers rather than GRA6 alone. Performing multilocus genotyping would result in more accurate genotype characteristics than a single gene.

## Conclusion

According to the results of the present study, the prevalence of toxoplasmosis in pregnant women in Urmia is similar to its prevalence in other areas in the northwest of the country, and despite the low prevalence of acute infection, due to possible serious complications on the fetus, it should not be ignored. Living in rural areas, contact with cats, contact with the soil, and not washing vegetables with vegetable disinfectants can increase the risk of toxoplasmosis. In the present study isolates carrying the GRA6 allele of Lineage I were identified in pregnant women in West Azerbaijan Province; however It is necessary to examine more samples for evaluation of *Toxoplasma* genotypes.

## Data Availability

The datasets, raw or analyzed will be available from the corresponding author on reasonable request.
